# Functional analysis of the actin-binding protein, tropomyosin 1, in neuroblastoma

**DOI:** 10.1038/sj.bjc.6601201

**Published:** 2003-08-26

**Authors:** M L Yager, J A I Hughes, F J Lovicu, P W Gunning, R P Weinberger, G M O'Neill

**Affiliations:** 1 The Oncology Research Unit, The Children's Hospital at Westmead, Locked Bag 4001, Westmead 2145, NSW, Australia; 2 Department of Anatomy and Histology, The University of Sydney, Sydney 2000, NSW, Australia; 3 Discipline of Paediatrics and Child Health, Faculty of Medicine, The University of Sydney, Sydney, NSW, Australia

**Keywords:** tropomyosin, actin, neuroblastoma, cytoskeleton

## Abstract

Tropomyosin 1 (TM1) is downregulated in a number of transformed cell types, and exogenous expression of TM1 can restore actin organisation and reverse cellular transformation. We find that TM1 is also downregulated in human neuroblastoma cell lines, correlating with increasing malignancy. However, exogenous TM1 does not restore actin cytoskeleton organisation in neuroblastoma cells.

Disorganisation of the actin cytoskeleton is a characteristic feature of transformation (reviewed in Pawlak and Helfman, 2001) and the potential to reinstate normal cytoskeletal structure and function represents a promising target for cancer therapy. Tropomyosins (TMs) contribute to actin cytoskeleton integrity by binding and stabilising actin filaments (Lin *et al*, 1997; Gunning *et al*, 1998), and decreased expression of tropomyosin 1 (TM1) is characteristic of cellular transformation in a number of cell types (Hendricks and Weintraub, 1981; Cooper *et al*, 1985; Bhattacharya *et al*, 1990; Masuda *et al*, 1996; Wang *et al*, 1996; Hughes *et al*, 2003). Exogenous TM1 expression can reverse transformation, suggesting a direct role for TM1 in transformation (Prasad *et al*, 1993; Braverman *et al*, 1996; Shah *et al*, 2001) and, conversely, enforced downregulation of TM1 causes the acquisition of transformed characteristics (Boyd *et al*, 1995). These observations have led to the proposal that TM1 may be a tumour suppressor (Prasad *et al*, 1999).

Cell lines derived from human neuroblastoma tumours are grouped according to three characteristic phenotypes that reflect changes in the underlying actin cytoskeleton. The three morphological types, substrate (S), intermediate (I) and neuroblastic (N), represent a decreasing order of cell size and substrate adhesion (Biedler *et al*, 1973; Ross *et al*, 1995) and a corresponding conversion to anchorage independence and tumorigenicity (Spengler *et al*, 1997). Notably, exogenous expression of the actin-binding protein actinin-4 in I-type cells reverted the cells to an S-type morphology (Nikolopoulos *et al*, 2000). This suggests that the actin cytoskeleton of neuroblastomas may be responsive to modulation by actin-binding proteins.

To date, the effect of TM1 expression on actin cytoskeleton organisation has primarily been studied in oncogene-transformed rat fibroblasts and little is known about the potential for TM1 to restore cytoskeletal structure and function in human cancer. Considering the well-described correlation between morphological characteristics and tumorigenicity in neuroblastoma, we have chosen to investigate the function of TM1 in cells derived from human neuroblastomas. We show that TM1 expression is downregulated in I- and N-type neuroblastoma cell lines and inversely correlates with increased expression of the prognostic indicator N-Myc. However, exogenous expression of TM1 in these cell lines does not alter either cellular morphology or the actin cytoskeleton organisation. Therefore, expression of TM1 alone is insufficient to restore normal cytoskeletal structure and function to the neuroblastoma cell lines tested.

## MATERIALS AND METHODS

### Antibodies and plasmids

Rabbit polyclonal *α*-f/9d antibodies that recognised TM1, and other TMs, have been previously described (Schevzov *et al*, 1997). Anti-*α*-tubulin was from Sigma (MO, USA), anti-N-myc was purchased from Oncology Research Products (CA, USA) and HRP-conjugated secondary anti-mouse and anti-rabbit antibodies were purchased from Amersham Pharmacia Biotech (UK). The TM1 expression construct (pTM1) consists of the 852 bp TM1 coding sequence inserted into the *Sal*I and *BamH*I coding sites of modified pEGFP-N1 (Clontech, CA, USA) that has the GFP coding sequence excised.

### Cell culture and transfection

The three neuroblastoma cell lines, SH-EP (S-type), BE(2)-C (I-type) and IMR32 (N-type), were routinely cultured in Dulbecco's modified Eagle's medium with 10% fetal bovine serum at 37°C with 5% CO_2_. Cultures of IMR32 and BE(2)-C cells were transfected with either pTM1 or empty vector using Lipofectamine 2000 as per the manufacturer's protocol (Gibco BRL, NY, USA). Pools of stably transfected cells were selected and maintained in the presence of 0.5 mg ml^−1^ G418.

### Preparation of cell lysates and Western blot analysis

Total proteins were extracted in 0.1% SDS-RIPA buffer (50 mM Tris pH 7.4, 150 mM NaCl, 5 mM EDTA, 1% Nonidet P-40, 0.1% SDS and 1% sodium deoxycholate). Cells were fractionated into detergent-soluble and -insoluble components as described previously (Polte and Hanks, 1997). Briefly, adherent cells were extracted for 2 min on ice with cytoskeletal stabilisation (CSK) buffer (0.3 M sucrose, 0.5% Triton X-100, 100 mM PIPES pH 6.8, 100 mM KCl, 1 mM CaCl_2_, 2.5 mM MgCl_2_, 50 mM NaF plus 1 mM sodium orthovanadate, 1 mM PMSF, 1 *μ*g ml^−1^ leupeptin and 1 *μ*g ml^−1^ aprotinin). Soluble proteins were aspirated and the remaining insoluble proteins extracted with 0.1% SDS-RIPA buffer. Loosely adherent cells were first collected by centrifugation, then incubated on ice in CSK buffer for 2 min and the insoluble proteins extracted as above. Volumes of corresponding soluble and insoluble samples were equalised prior to gel analysis.

Total proteins were separated by SDS–PAGE gel electrophoresis in 15% low bis-acrylamide 10 cm × 8 cm minigels (29.7% (wv^−1^) acrylamide, 0.3% (wv^−1^) bis-acrylamide stock solution). Western blots of separated proteins were probed with primary antibodies and bound antibodies detected using HRP-conjugated secondary antibodies and chemiluminescence was performed using the manufacturer's protocol (NEN Dupont, MA, USA).

### Immunofluorescence

Cells on coverslips were fixed with 4% paraformaldehyde for 15 min at room temperature, then permeabilised with 0.2% Triton X-100 for 5 min. Cells were then stained with TritC-phalloidin or with anti-*α*-f/9d followed by Alexa488-labelled donkey anti-rabbit antibodies from Jackson Immnuoresearch Laboratories (PA, USA). Immunofluorescently labelled cells were visualised using an Olympus BX50 fluorescence microscope (Tokyo, Japan), and photographed using a SPOT II CCD camera (Diagnostic Instruments Inc., MI, USA). Images were optimised for brightness and resolution by adjusting the histogram range and running a sharpen filter using ImagePro Plus software (Media Cybernetics, MD, USA).

## RESULTS

**Figure 1:**
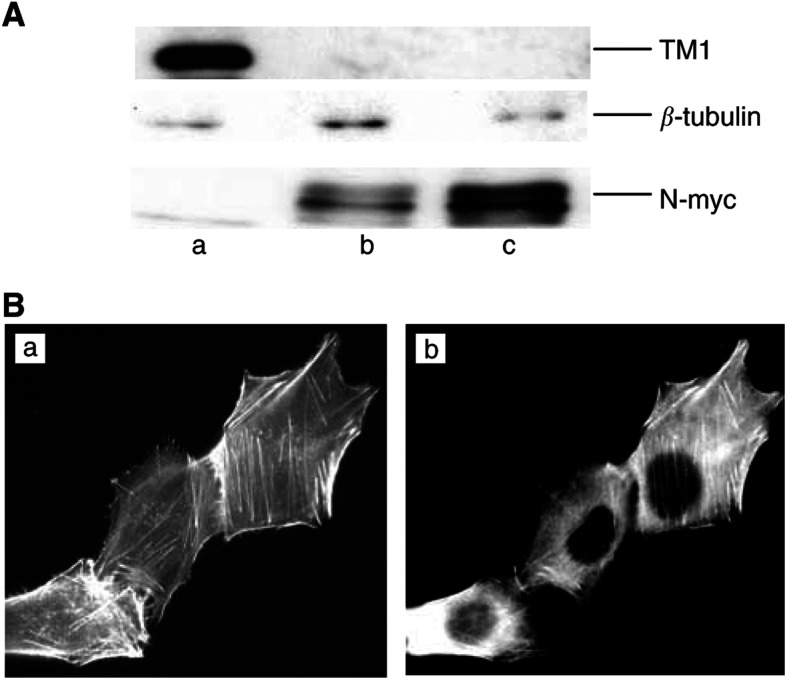
TM1 expression is downregulated in more malignant neuroblastomas. (**A**) Western blots of total protein extracts from SH-EP (a), IMR32 (b) and BE(2)-C (c). Equivalent concentrations of total protein were loaded for each cell line. Blots were probed with *α*-f/9d to detect TM1, anti-*β*-tubulin as a loading control and anti-N-myc antibodies, as indicated. (**B**) SH-EP cells were grown on coverslips and stained with TritC-phalloidin (a) or *α*-f/9d (b).

**Figure 2:**
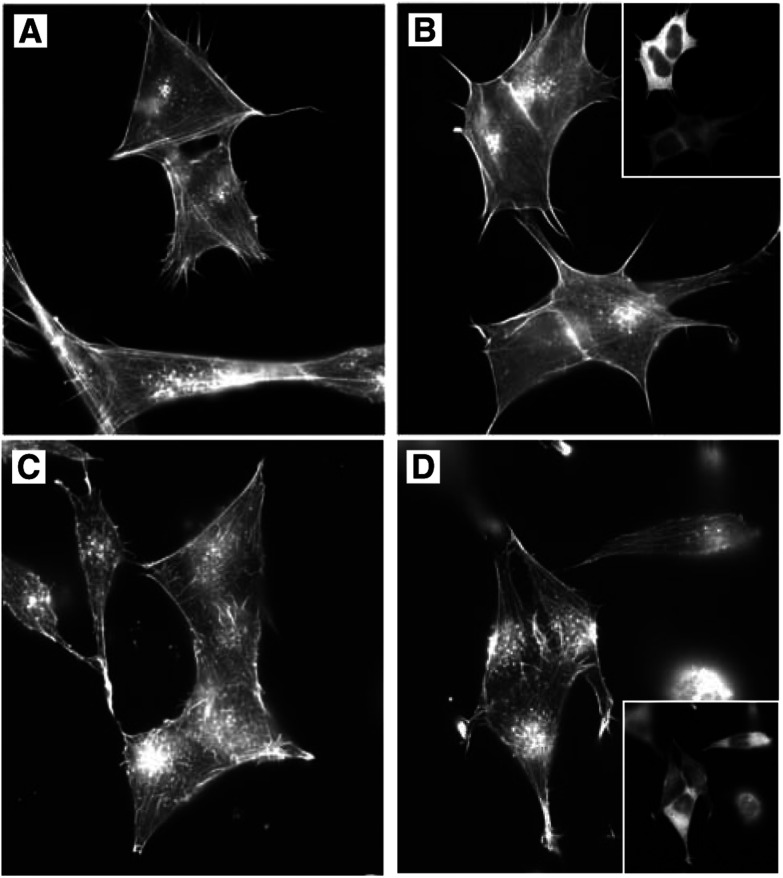
Exogenous TM1 expression does not alter the actin cytoskeleton. Vector (**A,C**) and TM1 (**B,D**) transfected IMR32 (**A,B**) and BE(2)-C (**C,D**) cells were grown on coverslips. Cells were then stained with TritC-phalloidin to detect actin microfilaments. Panels (**B,D**) show the same cells stained with *α*-f/9d to detect cells expressing TM1. The percentage of TM1-transfected cells calculated to be TM1-positive with *α*-f/9d staining was 36% IMR32 cells and 67% BE(2)-C cells.

The expression pattern of the putative tumour suppressor TM1 in neuroblastoma cells was determined by probing Western blots of protein extracts from each cell line with the *α*-f/9d antibody. The results show that the S-type SH-EP cells are TM1-positive, while the I-type BE(2)-C cells and the N-type IMR32 cells have no detectable TM1 expression (Figure 1A

). As predicted based on their morphological characteristics, the TM1 positive SH-EP cells have discernible actin stress fibres (Figure 1Ba), while the BE(2)-C and IMR32 cells both lack well-organised parallel bundles of stress fibres (Figure 2A and C

). Correspondingly, there is a clear filamentous tropomyosin staining pattern in the SH-EP cells (Figure 1Bb). Interestingly, the absence of TM1 expression correlates with increased N-myc expression (Figure 1A), a marker of rapidly growing, metastatic neuroblastomas (Goodman *et al*, 1997). Therefore, similar to observations in tumours derived from other cell types, loss of TM1 correlates with more malignant neuroblastoma cell types.

Following the observation that TM1 expression is absent in the cell lines with poorly organised cytoskeletons, we wished to determine whether exogenous expression of TM1 could restore actin organisation into stress fibre bundles in these cell lines. Stable pools of BE(2)-C and IMR32 cells transfected with either TM1 or empty vector control were prepared. Phalloidin staining of transfected cells shows that the actin cytoskeleton organisation in the TM1 transfectants is indistinguishable from that of the vector transfectants (Figure 2, compare A with B and C with D). This is supported by our observation that the TM1 transfectants have unchanged morphology. We further note that TM1 expression had no measurable effect on the growth rate of transfected cells (data not shown). Together, these results suggest that exogenous TM1 had no effect on the organisation of the actin cytoskeleton in the BE(2)-C and IMR32 cells.

**Figure 3:**
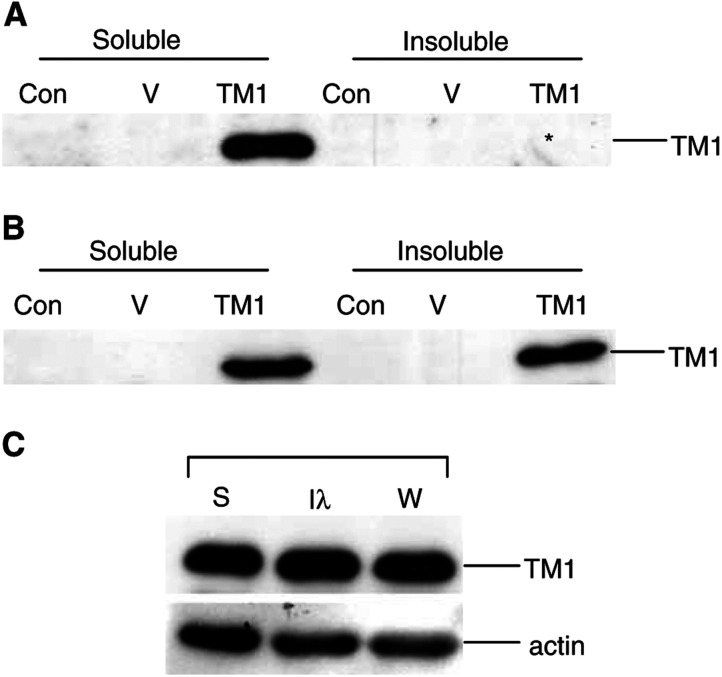
Exogenous TM1 localises to both the detergent-insoluble- and -soluble-fractions. Equal volumes of detergent-soluble and -insoluble fractions of control (C), vector-transfected (V) and TM1-transfected (TM1) cells were loaded into each lane of an SDS–polyacrylamide gel. Western blots were probed with *α*-f/9d to detect TM1 as indicated. (**A**) Lysates from IMR32 cells and (**B**) Lysates from BE(2)-C cells. ^*^Note that after a longer exposure, TM1 can be detected in the insoluble fraction of the IMR-32 TM1-transfectants. (**C**) Fractionated extracts from SH-EP cells showing the detergent-soluble fraction (S), the insoluble fraction (I) and the whole-cell lysate (W). Western blots of lysates were probed with *α*-f/9d and actin, as indicated.

It was possible that the lack of TM1 effect on actin organisation may have been due to an inability of exogenous TM1 to incorporate into stress fibres, therefore preventing TM1-mediated stress fibre stabilisation. Therefore, we assessed TM1 subcellular localisation. High cytosolic levels of the exogenous protein prevented the analysis of TM1 localisation at stress fibres by immunofluorescence analysis. Instead, cells were fractionated into detergent-soluble and -insoluble pools and the distribution of TM1 was assessed. The results demonstrate that exogenous TM1 associates with both the detergent-soluble and -insoluble fractions, suggesting that at least some of the exogenous TM1 is incorporating in the insoluble actin cytoskeleton compartment (Figure 3A and B

). We note that the distribution of the exogenous TM1 is similar to that of endogenous TM1 in the SH-EP cell line, where approximately equivalent levels of TM1 are seen in the soluble and insoluble fractions (Figure 3C). Therefore, the failure of exogenous TM1 to stimulate stress fibre formation is not due to inability of the TM1 to incorporate into the existing actin stress fibres.

## DISCUSSION

The downregulation of TMs and other actin-binding proteins is a common feature in transformed cell populations (Pawlak and Helfman, 2001) and we now confirm that this is also true in neuroblastoma cell lines. Our studies suggest that TM1 cannot act alone to restore the transformed phenotype. In the current study, we observed loss of not only TM1, but also TM2 and TM3 (data not shown), and the absence or loss of function of other TMs may inhibit the normal function of the exogenously added TM1. It is likely that the complement of actin binding protein expression in each tumour or cell type will ultimately determine our ability to restore normal actin cytoskeleton structure and function.

In a recent study, exogenous TM1 inhibited anchorage-independent growth of MCF7 breast cancer cells (Mahadev *et al*, 2002). In contrast, our study suggests that TM1 cannot restore normal actin cytoskeleton function to neuroblastoma cell lines. There are a number of possible explanations for the differences observed between the two cell types. In previously reported experimental systems, cells transformed with a single agent can be reverted by the introduction of TM1 alone (Prasad *et al*, 1993, 1999); therefore, it is possible that the multistep mutations that cause human cancer mean that replacing a single cytoskeletal protein will be insufficient to restore normal function in many cancers. Alternatively, the function of TM1 in different cell types may determine the effects of restoring TM1 expression in cancer cells. The expression of TM1 has been shown to be developmentally regulated in the brain (Hughes *et al*, 2003), suggesting that this isoform may play a role in specific differentiated tissues. As a specific cell type loses differentiated characteristics, such as is seen in the development of cancer, it may also consequently lose responsiveness to TM1 function. If this is the case, we can expect that cells of different origin, for example epithelial-derived breast cancer *vs* neural crest-derived neuroblastoma, will be differentially responsive to the effects of restored TM1 expression. Our data demonstrate that there is cell-type specificity to the ability of TM1 to restore normal cytoskeletal function. Therefore, the efficacy of this molecule as a target for reverse-transformation of cancer cells will need to be determined for each individual cancer type.

It is clear that the cytoskeleton contributes to many aspects of cancer progression. By appropriately targeting the cytoskeleton, we may be able to stimulate reversion of cancer cells. Further, the demonstrated role of the cytoskeleton in normal cellular function, including the systematic cellular events executed during apoptosis (Pawlak and Helfman, 2001), suggests that a better understanding of the molecular composition of the cytoskeleton may help overcome resistance to currently used therapies and aid in the design of more efficacious therapies.
